# Structural Characterization of the CD44 Stem Region for Standard and Cancer-Associated Isoforms

**DOI:** 10.3390/ijms21010336

**Published:** 2020-01-03

**Authors:** Kun-Lin Chen, Deng Li, Ting-Xuan Lu, Shu-Wei Chang

**Affiliations:** 1 Department of Engineering Science and Ocean Engineering, National Taiwan University, Taipei 10617, Taiwan; pen39251099@gmail.com; 2 Department of Civil Engineering, National Taiwan University, Taipei 10617, Taiwan; uplideng@gmail.com; 3 Department and Graduate Institute of Pharmacology, National Taiwan University, Taipei 10617, Taiwan; tingxuanlu19@gmail.com

**Keywords:** CD44, CD44v6, receptor tyrosine kinase (RTK), metastasis, tumor progression, β-sheets, molecular dynamics simulations

## Abstract

CD44 is widely expressed in most vertebrate cells, whereas the expression of CD44v6 is restricted to only a few tissues and has been considered to be associated with tumor progression and metastasis. Thus, CD44v6 has been recognized as a promising prognostic biomarker and therapeutic target for various cancers for more than a decade. However, despite many experimental studies, the structural dynamics and differences between CD44s and CD44v6, particularly in their stem region, still remain elusive. Here, a computational study was conducted to address these problems. We found that the stem of CD44s adopted predominantly two conformations, one featuring antiparallel β-sheets and the other featuring parallel β-sheets, whereas the stem of CD44v6 adopted mainly one conformation with relatively highly suppressed β-sheet contents. Moreover, Phe215 was found to be essential in the β-sheets of both CD44s and CD44v6. We finally found intramolecular Phe215–Trp224 hydrogen-bonding interactions and hydrophobic interactions with Phe215 that cooperatively drove conformational differences upon the addition of the v6 region to CD44. Our study elucidated the structural differences between the stem regions of CD44s and CD44v6 and thus can offer useful structural information for drug design to specifically target CD44v6 in promising clinical applications.

## 1. Introduction

CD44 is a member of the cartilage link protein family, with hyaluronan (HA) as its primary ligand [1,2]. CD44 is a type I transmembrane receptor protein that engages in many cellular processes, such as regulating cell–cell and cell–matrix adhesion and controlling cell proliferation, differentiation, migration, and survival [3]. CD44 proteins have a common structure consisting of four major parts: The extracellular HA-binding domain (HABD), the stem region, the transmembrane segment, and the cytoplasmic region [4]. The HABD confers binding capability to CD44 mainly for HA, but also for collagen, laminin, and fibronectin [5,6,7].

CD44 proteins are encoded by a single and highly conserved gene consisting of 20 exons [8]. Exons 1–5, 16–18, and 20 encode the smallest, the standard, and the hematopoietic isoform (“CD44s”), which is ubiquitously expressed in many vertebrate cells, including leukocytes, endothelial cells, neurons, and fibroblasts [4,9,10]. Ten variant exons, 6–15 (commonly called “v1–v10”), which are in the middle of the CD44 gene, can be alternatively spliced, yielding a wide variety of CD44 variant (CD44v) isoforms with different functions and structures in the stem region [8,11]. The heterogeneity of CD44 isoforms can be increased through decoration with *N*-glycans, *O*-glycans, and glycosaminoglycans, such as heparan sulfate and chondroitin sulfate [12,13,14,15].

Unlike CD44s, the expression of CD44v isoforms is restricted to only a few tissues [16,17]. CD44v isoforms have attracted keen interest because of their expression in a majority of tumors and the induction of the metastatic phenotype [9,18,19]. There is now abundant evidence for the crucial role of CD44v expression in the aggressive stages of various cancers as well as for its expression in cancer-initiating cells (also known as cancer stem cells) [20,21,22,23,24,25]. Among CD44v isoforms, CD44 variant 6 (CD44v6) plays a major role in cancer progression because its expression level correlates with a poor prognosis in many cancers, including colorectal cancer, lung cancer, hepatocellular carcinoma, prostate cancer, pancreatic cancer, endometrial cancer, and head and neck squamous cell carcinoma [26,27,28]. Furthermore, a recent study identified that CD44v6 is a marker of cancer stem cells in colon cancer and accounts for the metastatic phenotype of the tumors [29]. Therefore, CD44v6 is considered a promising prognostic biomarker and therapeutic target for various cancers [26,30,31,32]. CD44v6 proteins contain an exon v6-encoded region that can interact with the other major cytokines produced by the tumor microenvironment and with receptor tyrosine kinases (RTKs), such as c-Met, Ron, and vascular endothelial growth factor receptor 2 (VEGFR-2): Therefore they function as coreceptors, which plausibly explains how CD44v6 drives tumor progression and metastasis [33,34,35]. An alanine-scanning mutational analysis was used to determine that three amino acids (AAs) in the v6 sequence are required for CD44v6 to act as a coreceptor for RTKs [36].

Numerous experimental and computational studies on CD44 have been conducted. In addition to numerous experiments concentrating on its pathological functions and related signaling pathways [21,37], much work has focused on the molecular-level details of its structure and dynamics, particularly on HABD [38,39,40,41,42]. Several CD44 structures identified using X-ray crystallography or nuclear magnetic resonance (NMR) imaging have been deposited to the Protein Data Bank (PDB) [43,44,45,46]. Understanding of the nature of CD44 and its isoforms has considerably progressed. However, to the best of our knowledge, molecular-level insight into the structure and dynamics of CD44s and CD44v6 is largely missing because of the lack of suitable experimental techniques that can capture the structural information of the dynamic region within a protein at fine spatial and temporal resolution [47]. Because of the lack of information regarding the structural differences between CD44s and CD44v6, achieving a structure-based rational drug design remains difficult, which slows down the development efficiency of strategies to specifically target CD44v6 in clinical applications [48,49].

**Figure 1:**
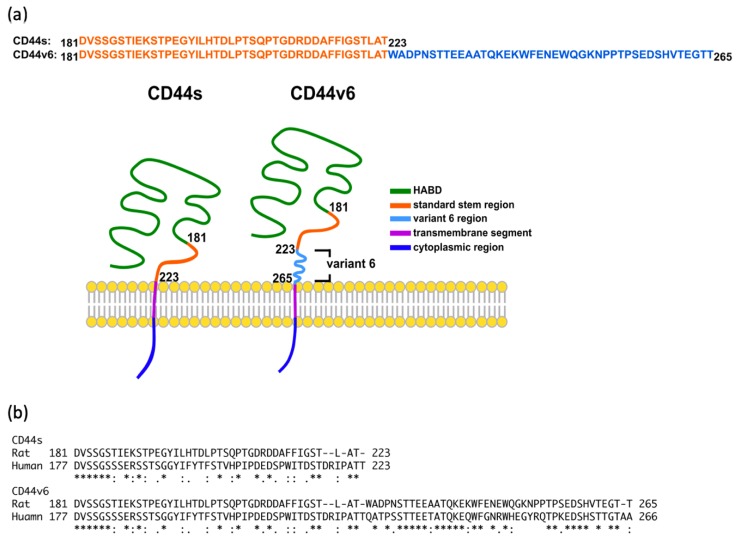
(**a**) Sequence comparison of CD44s and CD44v6 isoform of rat. Different regions are shown in different colors. (**b**) Sequence comparison of human and rat. An * (asterisk) indicates positions which have a single, fully conserved residue. A: (colon) indicates conservation between groups of strongly similar properties. A. (period) indicates conservation between groups of weakly similar properties.

Therefore, in the present study, we aimed to explore the conformational dynamics of the stem region of CD44s and CD44v6 and to characterize the differences between them. We conducted all-atom explicit-solvent molecular dynamics (MD) simulations to explore the structure of the CD44s and CD44v6 stem regions, which is the only region that differs between these two isoforms in terms of AA sequences (Figure 1a). MD simulation uses physics-based force fields and is a computational approach that can provide valuable insight into protein dynamic characteristics at an atomic level, which is extremely difficult to achieve with any experimental technique [47,50,51]. Consequently, MD simulation serves as a powerful tool to complement experimental techniques for characterizing the conformational ensembles of the CD44s and CD44v6 stem regions.

Since the CD44s and CD44v6 stem regions have not been structurally solved using X-ray crystallography or NMR imaging, we generated 3D models of these proteins by using the threading method. Long-timescale MD simulations were employed to sample their conformations. The sampled conformations were analyzed for further characterization. We termed the “CD44s stem region” the “standard stem region”, and the “CD44v6 stem region” is composed of the “standard stem region” and “variant 6 region”. The AA sequences of the standard stem region of CD44s and CD44v6 were exactly the same (Figure 1a). In previous studies, rat models were widely used in CD44-related research. Figure 1b provide the sequence comparison of human and rat. Previous studies have successfully proved the significance of CD44v6 in rat models [52,53,54]. Thus, we began our study with rat sequence.

## 2. Results

### 2.1. CD44s Stem Region Adopted Predominantly Two Conformations, Whereas CD44v6 Mainly Adopted One

Due to the low sequence similarity (<30%) of solved proteins in the PDB (Figure S1), the threading method was employed to obtain a rough model of target sequences instead of homology modeling [55,56]. After sending the protein sequence to the I-TASSER, a popular server system that develops 3D models by using multiple threading alignments [57], five rough 3D models were obtained. The best C-scores (the confidence score of the predicted model) of the first two models for the CD44s stem region were −2.04 and −4.10, and those for the CD44v6 stem region were −3.31 and −3.69. The four models were further assessed using VERIFY3D to assess the protein models with three-dimensional profiles: 100% and 69.77% of the residues in the two models of the CD44s stem region exhibited an average 3D–1D score of >0.2, whereas percentages of 48% and 29% were obtained for the CD44v6 stem region. The models with lower percentages were discarded.

**Figure 2:**
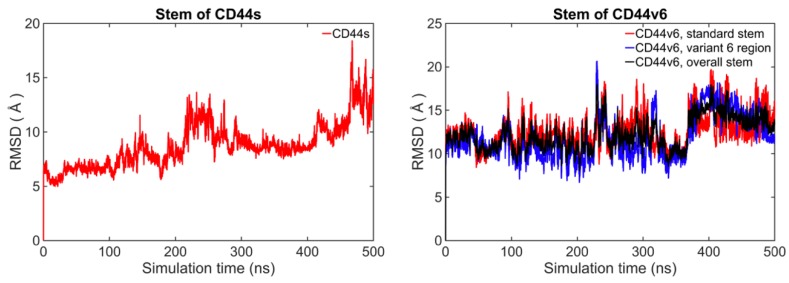
The RMSD comparison between the CD44s stem region and the CD44v6 stem region based on average structure.

The selected models of the CD44s and CD44v6 stem regions were then energy-minimized. Subsequently, two atomistic MD simulations were performed separately for a long time duration of 500 ns on the two systems—the stem region of CD44 and the stem region of CD44v6—to explore their conformational dynamics and structural properties. The root mean square deviations (RMSDs) were monitored over a 500 ns trajectory to view the extent of conformational sampling overall. The RMSDs relative to average structure are shown in Figure 2. The RMSDs of the standard stem region for CD44s and CD44v6 both mainly ranged from 6 Å to 16 Å. A large fluctuation in the RMSD value of the backbone atoms for both cases indicated dynamic behavior of the standard stem region for both CD44s and CD44v6. Notably, no considerable variation in the RMSD of the standard stem region backbone of both CD44s and CD44v6 was observed from 330 to 470 ns.

**Figure 3:**
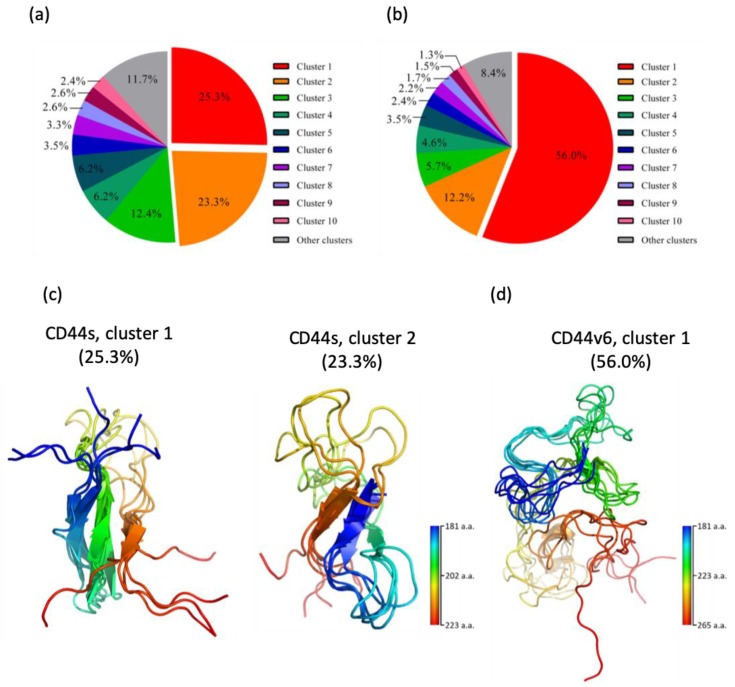
Clustering analysis of the CD44s and CD44v6 stem regions. (**a**) Population distribution of each cluster of the CD44s stem region. (**b**) Population distribution of each cluster of the CD44v6 stem region. (**c**) Structures of the most populated clusters of the CD44s stem region, cluster 1 and cluster 2. (**d**) Structures of the most populated cluster of the CD44v6 stem region, cluster 1. Ensemble structures are rainbow-colored from blue at the *N*-terminus to red at the *C*-terminus.

To characterize the conformational ensemble sampled using MD for detailed investigation, we performed a clustering analysis on the standard stem region in the CD44s and CD44v6 systems. In this calculation, we classified the sampled structures into distinct clusters on the basis of their RMSD values with a cutoff of 8.5 Å. The population distributions of the first 10 most populated clusters are shown in Figure 3a,b. Through this analysis, we found two predominant conformations in our simulations of the CD44s stem region, with population distributions of 25.3% (cluster 1) and 23.3% (cluster 2), which together represented approximately 50% of all conformations. In contrast, a single major conformation was detected for the CD44v6 stem region, with 56.0% (cluster 1) of the population of all conformations. The structures obtained from the clustering analysis are presented in Figure 3c,d. It is apparent that the most populated clusters of the CD44s stem region, cluster 1 and cluster 2, were both mixed with loops and β-sheet secondary structures, whereas the most populated cluster of the CD44v6 stem region, cluster 1, exhibited much less β-sheet content. A significant structural difference between cluster 1 and cluster 2 in CD44s was the β-sheet arrangement. In cluster 1, β-sheets were arranged in an antiparallel manner consisting of three β-strands. In contrast, in cluster 2, β-sheets were arranged in a parallel manner and consisted of two β-strands. These observations are in agreement with the calculation indicating that cluster 1 had a higher population than cluster 2 insofar as the antiparallel β-sheet was more abundant and energetically favored than the parallel β-sheet was [58,59]. Due to the aforementioned results, we selected the most populated clusters of the CD44s stem region (cluster 1 and cluster 2) and cluster 1 in the CD44v6 stem region for further analyses.

### 2.2. The Standard Stem Region of CD44v6 Was Much More Flexible than CD44s

**Figure 4:**
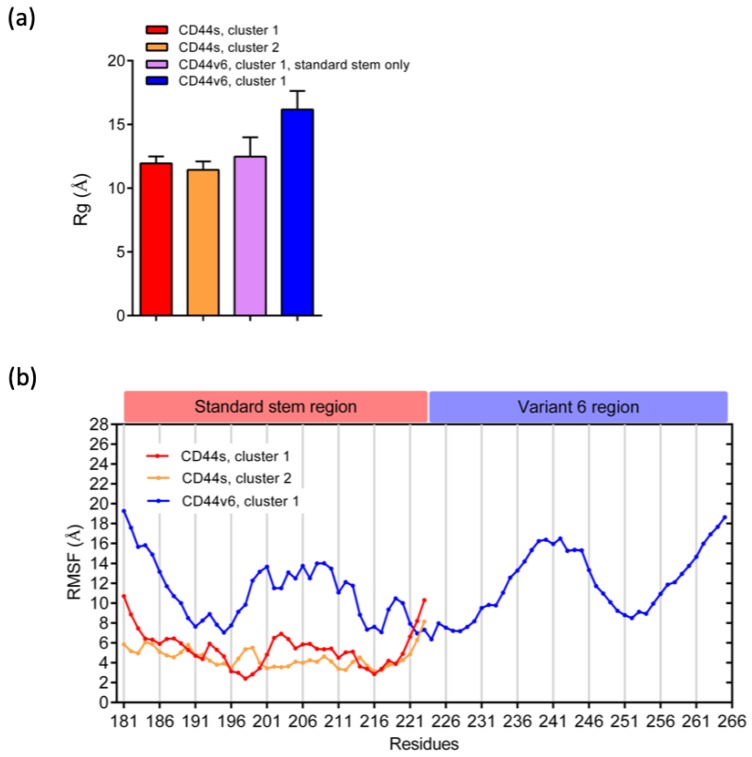
Analysis of (**a**) the radius of gyration (Rg) and (**b**) the root mean square fluctuation (RMSF) for the Cα atoms of the most populated clusters.

We calculated the radius of gyration (Rg) to understand the levels of compaction of the CD44s and CD44v6 stem regions. Rg is defined as the mass-weighted root mean square distance of a collection of atoms from their center of mass [60]. Therefore, this analysis could provide the overall dimensions of the protein. The Rg values of the standard stem region in CD44s and CD44v6 were quite similar and were all approximately 12 Å, as shown in Figure 4a. Although the conformational differences between CD44s and CD44v6 in the standard stem region were evident, as mentioned previously, the Rg results indicated that the v6 region did not affect the standard stem region by markedly changing its size. However, considering the overall CD44v6 stem region, we could see an increase in the Rg value to an average of 16.17 Å, as expected due to an increase in the number of AAs.

To determine whether the addition of v6 affected the dynamic behavior of the standard stem on particular residues, we calculated the Cα root mean square fluctuation (RMSF) of their stem region to measure the flexibilities of each residue (Figure 4b). The CD44v6 stem region exhibited an overall higher RMSF than did the CD44s stem region, suggesting a perturbation effect upon the addition of v6 to the stem region. Notably, for cluster 1 of CD44s, two minimums at the residue regions of 196–200 and 214–217 were observed, indicating the low flexibility of these two regions. In contrast, the overall RMSF of CD44s cluster 2 was relatively flat and slightly lower than that of cluster 1. In CD44v6 cluster 1, local minimums were clearly observed in the residue regions of 190–196, 215–217, 222–224, and 250–254, indicating the low flexibility of these regions. Taken together, these results demonstrated that when v6 was present, there was a gain in flexibility and an altered fluctuation of residues in the standard stem region without an effect on the overall compactness.

### 2.3. Phe215 Displayed a High Tendency to Form β-Strands in CD44s and CD44v6, Whereas the CD44v6 Stem Region Exhibited Fewer β-Sheets than CD44s Did

**Figure 5:**
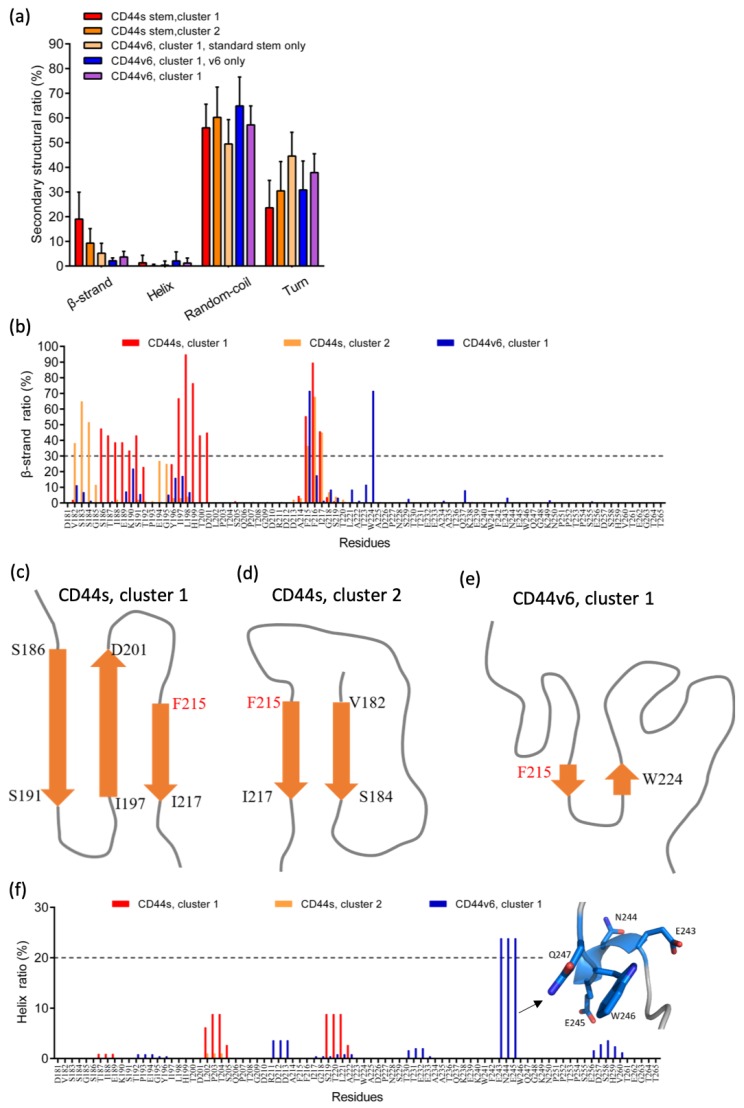
Analysis with secondary structure and regular secondary structure (β-sheet and helix) composition on residue base. (**a**) Average ratio of each secondary structure (including helix, β-sheet, coil, and turn) for all residues and (**b**) β-sheet ratio as a function of amino acid residue. Illustrations of the β-strand arrangements of (**c**) the CD44s stem region, cluster 1; (**d**) the CD44s stem region, cluster 2; and (**e**) the CD44v6 stem region, cluster 1. (**f**) Helix ratio as a function of amino acid residue.

To quantitatively investigate the observed β-sheet structures, we calculated the β-sheet average ratio for all residues and the residue-based composition ratio of CD44s and CD44v6 by using the protein secondary structure assignment algorithm STRIDE [61]. The ratio of the other secondary structures (helix, coil, and turn) was also analyzed, as shown in Figure 5a. The standard stem region of CD44v6 cluster 1 exhibited much fewer β-sheets compared to CD44s, as shown in Figure 3c,d. The β-sheet contents for cluster 1 and cluster 2 of CD44s were 19.0% and 9.3%, respectively, and the ratio was reduced to 5.2% with the addition of the v6 region. In random-coil contents, a similar tendency of a decrease in the ratio upon the addition of the v6 region was observed. In contrast, the turn contents for cluster 1 and cluster 2 of CD44s were 23.6% and 30.5%, which increased to 44.6% when the v6 region was present. All of the ratios of the helical structure except for the v6 region were considerably small (<1.5%). These results indicated that the introduction of v6 could considerably inhibit β-sheet and coil formation and promote turn formation in the standard stem region.

Figure 5b shows the propensities of β-sheets as a function of AA residues. The residue-based β-sheet ratios displayed quite different distributions. In CD44s cluster 1, AAs 186–191, 197–201, and 215–217 indicated a strong tendency (>30%) to form β-sheets. We found that these regions favored the low fluctuated residues in the RMSF plot (Figure 4b), which allowed us to elucidate the structurally stable region in the conformation. The antiparallel β-sheet structure that consisted of the three β-strands is depicted in Figure 5c. In CD44s cluster 2, only AAs 182–184 and 215–217 exhibited >30% β-sheet tendency, and the two β-strands assembled into a parallel β-sheet, as depicted in Figure 5d. Although two regions formed β-sheet structures, the RMSF analysis did not indicate relatively low flexibility in these regions, as was apparent in CD44s cluster 1 and CD44v6 cluster 1. This behavior was observed because parallel β-sheets are generally less stable than antiparallel β-sheets [62]. In CD44v6 cluster 1, only Phe215 and Trp224 exhibited high β-sheet propensity (both 71.43%), together forming a substantially short antiparallel β-sheet, as depicted in Figure 5e. These two residues were located in the local minimum of the RMSF analysis, which explained the local structural stability. These analyses identified the crucial residues involved in the β-sheet structures that affected the local structure flexibility and were consistent with the RMSF investigation results.

Notably, the involvement of residue Phe215 in the β-sheet structure was conserved in both CD44s and CD44v6, implying a crucial role of Phe215 in the cooperativity of β-sheets. The high β-sheet propensity of phenylalanine has been observed in previous studies [63,64]. Notably, Phe215 was situated at the first position in all three β-sheets. In addition, although the overall helical contents were considerably low for all cases (Figure 5a), we found that Glu243, Asn244, and Glu245 within v6 exhibited a considerably higher helical tendency compared to the other residues, as shown in Figure 5f. These three residues were adjacent only to the three AAs 245EWQ247, which were identified by mutational analysis to be critical for the coreceptor function of CD44v6, as described in the introduction [36]. The results present a structural view around 245EWQ247 within the v6 sequence, which is essential for the collaboration between CD44v6 and RTKs, which induces the cancer metastatic process [33].

### 2.4. CD44s and CD44v6 Utilized Phe125 with Different Interaction Partners to Form Hydrogen Bonds (Hbonds) to Stabilize β-Sheets

**Figure 6:**
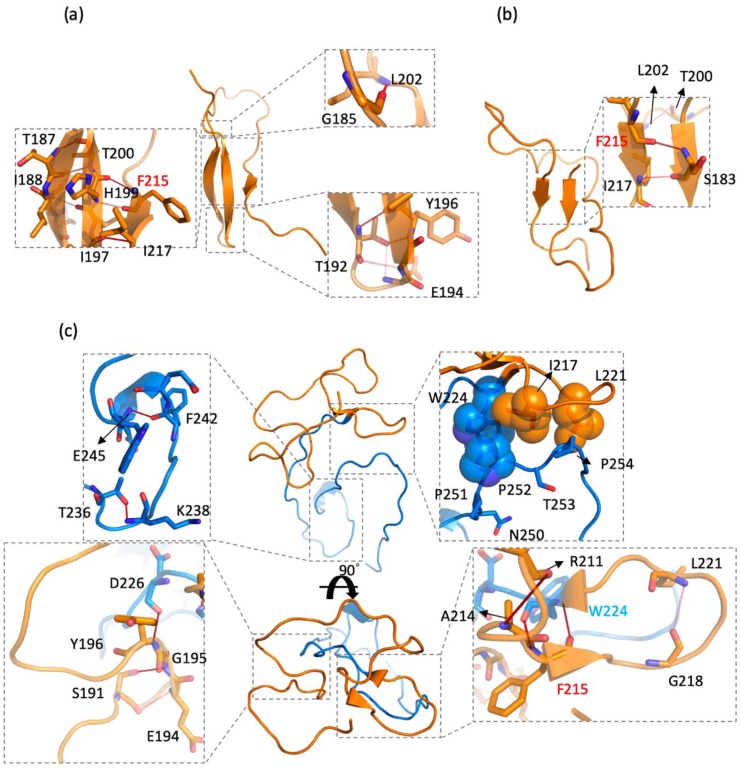
Detailed hydrogen bonds and intramolecular interaction networks: (**a**) the CD44s stem region, cluster 1 (also see Table S1); (**b**) the CD44s stem region, cluster 2 (also see Table S2); (**c**) the CD44v6 stem region, cluster 1 (also see Table S3). The standard stem region is colored orange, and the v6 region is colored blue. The key residues involved in interactions are shown in sticks. The key residues involved in the hydrophobic contacts with Pro252 are shown in spheres. Hydrogen bonds are depicted with red dash lines.

Hydrogen-bonding interactions have long been considered crucial in the stability of a protein structure [65,66]. To identify the key residues for hydrogen bonds (Hbonds), we have presented the Hbonds interaction pairs with an occupancy of >35% in CD44s and CD44v6 in Tables S1–S3. Among all of the residues, Phe215 was the only AA that formed Hbonds in CD44s and CD44v6, but with different interaction partners. The main chain of Phe215 formed persistent Hbonds with His199 (occupancy of 88.60%) in CD44s cluster 1, Ser183 (occupancy of 65.71%) in CD44s cluster 2, and Trp224 (occupancy of 75.40% and 63.10%). The strong occupancies of Phe215 in the Hbonds indicated the crucial role of the residue in stabilizing β-sheet structures, which was consistent with the aforementioned observations. Moreover, the results may suggest that Trp224 in the v6 region competed with His199 and Ser183 for the formation of Hbonds with Phe215, which caused the β-sheet structures, disrupting the standard stem region. A structural view of Hbond interactions is shown in Figure 6.

Although we performed an Hbonds analysis for both the side and main chains of all residues, a majority of persistent Hbonds pairs were between the main chains in both CD44s and CD44v6, revealing that backbone–backbone chain Hbonds were the main conformation stabilizing forces. As presented in Tables S1 and S2, more than half of the Hbonds facilitated β-sheet formation. In CD44s cluster 1 (Figure 6a), 6 out of 11 persistent Hbonds were involved in a β-sheet structure, whereas in CD44s cluster 2, two out of three Hbonds were involved (Figure 6b), indicating a major contribution of Hbonds in CD44s. Notably, in CD44v6 cluster 1 (Figure 6c), three persistent Hbonds between the standard stem region and the v6 region prevented the two regions from separating completely but allowed for individual activity in each region. Additionally, a set of stable Hbonds between Glu194 and Ser191, Gly195 and Ser191, and Tyr196 and Asp226 considerably contributed to the stabilization of the loop that encompassed AAs 191–196, as shown in Figure 6c, thus leading to less flexibility in AAs 191–196, as analyzed in the RMSF plot (Figure 4b).

In the RMSF plot, the cause of the region of AAs (250–254) in CD44v6 that appeared to be less flexible is still unknown, because there were no helixes, β-sheets, or even any persistent Hbonds in the region. To address this problem, we thoroughly investigated these five residues, 250NPPTP254. It was actually a proline-rich region, which is generally less flexible because proline exhibits a unique side chain that wraps around to form a covalent bond with the backbone, severely limiting backbone motility [67,68]. In addition, considering the hydrophobic side chain of proline, we calculated the hydrophobic interaction with Pro251, Pro252, and Pro254. However, persistent hydrophobic contacts (occupancy of >35%) were only formed with Pro252, which was 49.2% for Pro252–Ile217, 40.5% for Pro252–Trp224, and 38.1% for Pro215–Leu221. The hydrophobic interaction network with 250NPPTP254 is shown in Figure 6c.

### 2.5. Phe215 in CD44v6 Interacted with Surrounding Hydrophobic Residues More than CD44s, Leading to Becoming Blocked

**Figure 7:**
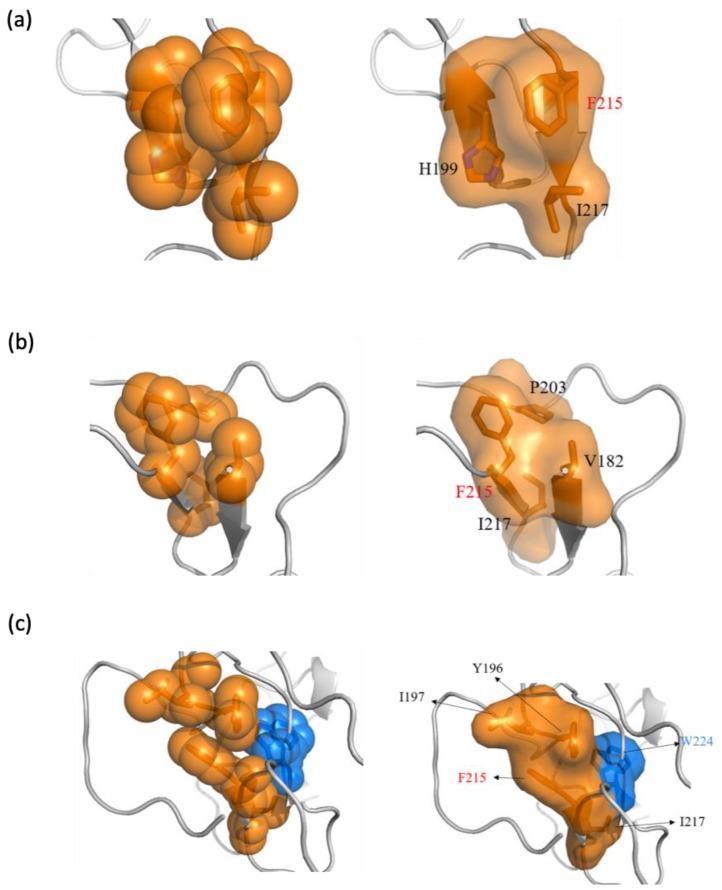
The hydrophobic and aromatic interaction network with the key residue Phe215 in (**a**) the CD44s stem region, cluster 1; (**b**) the CD44s stem region, cluster 2; and (**c**) the CD44v6 stem region, cluster 1. The residues involved in the hydrophobic and aromatic interactions are colored orange and are shown in sticks with spheres (left) and surfaces (right).

Due to the aromatic and highly hydrophobic side chain of phenylalanine, it is highly likely that hydrophobic and aromatic stacking interactions with Phe215 contribute to consolidating the structure [69]. Thus, we performed a hydrophobic/aromatic interaction analysis on Phe215. The identified hydrophobic and aromatic residues are listed in Table S4. The hydrophobic and aromatic interaction network with Phe215 is shown in Figure 7. The analysis revealed that, compared to Phe215 in CD44s, the Phe215 in CD44v6 interacted more with hydrophobic residues, two of which contained an aromatic ring, namely Tyr196 and Trp224. Notably, Trp224 in v6 not only interacted with Phe215 through the aforementioned strong main chain Hbonds but also through side chain aromatic interaction, reflecting the strong tendency of Trp224 to interact with Phe215.

To evaluate the contribution of hydrophobic and aromatic stacking interactions, we computed the interaction energy (sum of columbic and van der Waals energies) between the key residues in Table S4 and Phe215. As shown in Table 1, the Phe215 in CD44v6 exhibited the lowest average interaction energy of −2.476 kcal/mol, which was primarily contributed by the van der Waals effect. Thus, the interaction mode with the surrounding hydrophobic residues for Phe215 was more favored in CD44v6. We also computed the buried percentage of the solvent-accessible surface area (SASA) of Phe215, which is a measure of the AA surfaces exposed to the solvent. As shown in Table 2, the Phe215 in CD44v6 exhibited a higher buried SASA (70.96%) percentage than did Phe215 in CD44s because of shielding from water exposure by the surrounding hydrophobic residues. Taken together, these results reveal that both hydrogen bonding and hydrophobic interactions cooperatively drive the local interaction network around Phe215, leading to a conformational change in the stem region upon the addition of the v6 region.

## 3. Materials and Methods

### 3.1. Protein Model Preparation

Since the stem region of CD44s and CD44v6 have not been structurally characterized with NMR or X-ray crystallography, it was necessary to generate and validate 3D models of these proteins. In order to build a three-dimensional model of protein structures, amino acid sequences of the stem region of CD44s and CD44v6 were retrieved from the Uniprot database (P26051). The amino acid sequences for variant exon 6 were identified from previous studies [70,71]. The sequences for the stem region of CD44s and CD44v6 were 181DVSSGSTIEKSTPEGYILHTDLPTSQPTGDRDDAFFIGSTLAT223 and 181DVSSGSTIEKSTPEGYILHTDLPTSQPTGDRDDAFFIGSTLATWADPNSTTE EAATQKEKWFENEWQGKNPP TPSEDSHVTEGTT265 (the v6 region is underlined), respectively. The sequences were used as inputs for the Iterative Threading ASSEmbly Refinement (I-Tasser) server, which combines various techniques including threading, ab initio modeling, and structure refinement approaches [57,72,73]. I-TASSER produced five models for both of the sequences submitted, and only the first two models with the best C-scores for both stem regions were retained. The structural quality of the provided models were assessed using the Verify3D program (http://servicesn.mbi.ucla.edu/Verify3D/) [74,75].

### 3.2. Molecular Dynamics Simulations

The validated model of the stem region of CD44s and CD44v6 was subjected to all-atom explicit-solvent MD simulations using NAMD 2.13b2 [76] with a CHARMM36 force field [77,78,79,80]. Two systems were set up: One was the solvated stem region of CD44s, and the other was the solvated stem region of CD44v6. Both of the systems were solvated in a rectangular box of TIP3P water molecules using the solvate plug-in of visual molecular dynamics (VMD) [81], which extended at least 15 Å from the surface of the protein, and the systems were neutralized with NaCl using the autoionize plug-in of VMD. The salt (NaCl) concentration was set to 0.15 mol/L. Both of the systems were energy-minimized for 1000 steps followed by a 500 ns MD simulation performed at 310 K and 1 atm under the isothermal-isobaric (NPT) ensemble. The systems were simulated in periodic boundary conditions using the Langevin algorithm to maintain the temperature at 310 K and the Langevin piston Nose–Hoover method to keep the pressure constant at 1 atm. The particle mesh Ewald (PME) method with a grid size of less than 1 Å was used to calculate the electrostatic interactions [82]. The SHAKE algorithm was applied to constrain all of the covalent bonds with hydrogen, and the time step was set to 2 fs [83]. Van der Waals interactions were computed using a cutoff of 12 Å with a switching function starting at 10 Å. Nonbonded interactions were fully scaled starting with 1–4 bonded atoms. The first 50 ns over the simulation trajectory was treated as equilibration. The trajectory from 50 ns to 500 ns was recorded every 1 ns, and thus 450 frames were collected for analysis.

### 3.3. Data Analysis

Root mean square deviations (RMSDs) were calculated for the entire simulation trajectory with reference to their first frames for both proteins. Extracted protein snapshots were then clustered using the clustering tool in VMD at a RMSD cutoff value of 8.5 Å, based on backbone atoms of the standard stem region (i.e., AAs 181–223) for CD44s and CD44v6. The root mean square fluctuations (RMSFs) and radius of gyration (Rg) were calculated by using the coordinates of the Cα atoms. Secondary structural content was calculated by STRIDE [58] with the Timeline plug-in of the VMD package. The criteria to consider a hydrogen bond were a donor–acceptor distance less than 3.5 Å and a donor hydrogen–acceptor angle less than 45°. Hydrophobic and aromatic contacts were considered to exist if the distances between atoms in the side chains of hydrophobic/aromatic and hydrophobic/aromatic residues (Val, Leu, Met, Ile, Pro, Trp, Phe, His, and Tyr) were smaller than 5 Å. The solvent-accessible surface area (SASA) was measured with a spherical probe of 1.4 Å, mimicking a water molecule. The buried percentage of SASA was obtained by the formula (the buried SASA)/190.02 × 100%, where 190.02 is the fully exposed SASA of the side chain of phenylalanine in our calculation. All structural images were generated using PyMOL (PyMOL Molecular Graphics System, Version 1.3, Schrödinger, LLC).

## 4. Conclusions

Our results revealed that the differences in amino acid sequences between CD44s and CD44v6 led to different structures. By performing long-timescale all-atom MD simulations, we found that the stem region of CD44s adopted predominantly two conformations: (1) antiparallel β-sheets with a population of 25.3% and (2) parallel β-sheets with a slightly lower population of 23.3%. However, the CD44v6 stem region adopted mainly one conformation with a population of 56%, and the β-sheet contents were highly suppressed compared to the CD44s stem region. Notably, when v6 was present, there was a gain in flexibility and an altered fluctuation of residues in the standard stem region that did not influence overall compactness. It is worth noting that the insertion of other variants might also alter the CD44s stem region. The computational approached proposed in this work could be applied to resolve the molecular structures of other variants. We have also performed a molecular simulation with the insertion of v7 and the results confirm that the β-sheets contents were also highly suppressed (Figure S2 and Table S5 in Supplementary Materials).

**Figure 8:**
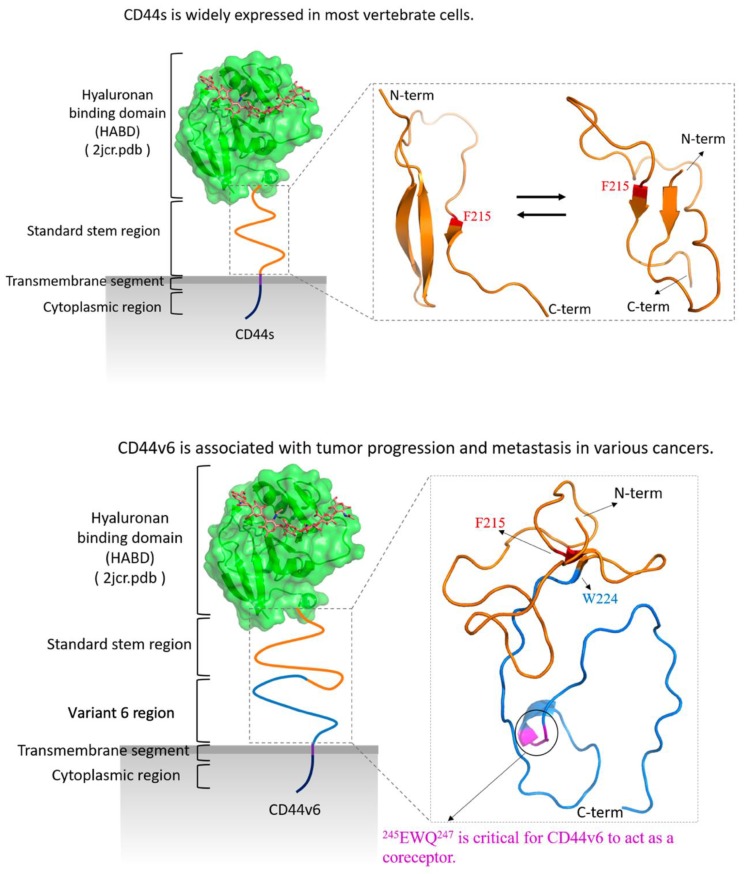
Schematic for the molecular structures and biological characteristics of CD44s (top) and CD44v6 (bottom). The structure of the HABD (hyaluronan-binding domain) was retrieved from the Protein Data Bank (PDB) (ID: 2JCR), and hyaluronan is shown in sticks. The identified key residue Phe215 is colored red in both CD44s and CD44v6. The standard stem region is colored orange, and the v6 region is colored blue. The amino acids (AAs) 245EWQ247, which are required for CD44v6 to act as the coreceptor for receptor tyrosine kinases (RTKs), including c-Met, Ron, and VEGFR-2, are colored magenta.

In addition, Glu243, Asn244, and Glu245 within v6 exhibited a relatively higher helical ratio than did the other residues. Their proximity to 245EWQ247 was identified as being essential for CD44v6 to act as a coreceptor. Notably, we found that Phe215 was the only residue that participated in the formation of β-sheets in both CD44s and CD44v6, highlighting its crucial role in stabilizing the structure. The residue Phe215 formed persistent Hbonds with either His199 or Ser183 in CD44s. However, Phe215 formed persistent Hbonds with Trp224 in CD44v6. The observations indicated that the residue Trp224 in the v6 region played a competing role against His199 and Ser183 to form strong Hbonds with Phe215, which led to a completely different arrangement of β-sheet structures. The interaction energy also indicated that the Phe215 in CD44v6 interacted with its surrounding hydrophobic residues more energetically and favorably than did the Phe215 in CD44s. Therefore, intramolecular Phe215–Trp224 hydrogen-bonding and hydrophobic interactions with Phe215 cooperatively drove the conformational differences upon addition of the v6 region to CD44 (Figure 8).

In summary, this is the first study that has reported molecular-level details of the stem regions of CD44s and CD44v6. The results obtained from this study will be useful for future studies on structure-based drug design to precisely target CD44v6 and yield promising clinical strategies.

## Figures and Tables

**Table 1:** Interaction energy (kcal/mol) between the side chain of Phe215 and the residues that were involved in the persistent hydrophobic contacts listed in Table S4. All of the values are presented as mean ± SD.

	van der Waals Energy	Electrostatic Energy	Interaction Energy
CD44s, cluster 1	−1.196 ± 1.058	−0.1625 ± 0.3979	−1.358 ± 1.243
CD44s, cluster 2	−1.534 ± 0.8957	0.1097 ± 0.2475	−1.424 ± 0.9442
CD44v6, cluster 1	−2.394 ± 1.445	−0.08138 ± 0.2759	−2.476 ± 1.485

**Table 2:** Solvent-accessible surface area (SASA) for the side chain of the Phe215 in CD44s and CD44v6. All values are presented as mean ± SD.

	SASA (Å2)	Buried Percentage (%)
CD44s, cluster 1	114.30 ± 31.24	39.85 ± 16.44%
CD44s, cluster 2	92.19 ± 31.64	51.48 ± 16.65%
CD44v6, cluster 1	55.18 ± 36.08	70.96 ± 18.99%

## Supplementary Materials

The following are available online at https://www.mdpi.com/1422-0067/21/1/336/s1.

## Abbreviations

HA	Hyaluronan
HABD	HA-binding domain
RTKs	Receptor tyrosine kinases
VEGFR-2	vascular endothelial growth factor receptor 2
NMR	Nuclear magnetic resonance
PDB	Protein Data Bank
MD	Molecular dynamics
RMSD	Root mean square deviation
Rg	Radius of gyration
RMSF	Root mean square fluctuation
SASA	Solvent-accessible surface area
