# Supplementary material for: Structural Characterization of the CD44 Stem Region for Standard and Cancer-Associated Isoforms
Source: Int J Mol Sci. 2020 Jan 3;21(1):336. doi: 10.3390/ijms21010336 (PMC6982006; doi:10.3390/ijms21010336)
Supplement: Supplementary file 1 [file ijms-21-00336-s001.pdf]

# Supplementary information

```
BLAST results
The best two hit comparison  obtained from BLAST

The symbol '#' means the atoms in the pdb file were not successfully
solved or missing.



for the standard stem region of CD44s

CD44s  :  181 DVSSGSTIEKSTPEGYILHTDLPTS-QPTGDRDDAFFIGSTLAT 223
similarity
4XGU_A :   83 EN--DLTINPSNIHVYKLHKDGPLS#############SQLWQ 126
                    **  *    * ** * * *              * *
11/43 = 25.58%

CD44s  :  181 DVSSGSTIEKSTPEGYILHTDLPTSQPTGDRDDAFFIGSTLAT 223
2DK1_A :    3 SGSSGRWVEGITSEGYHYYYDLISGASQWEKPEGFQGDLKKTS 45
                ***   *  * ***    **            *
11/43 = 25.58%


for the stem of CD44v6
CD44v6 : 181 DVSSGSTIEKSTP--
EGYILHTDLPTSQPTGDRDDAFFIGSTLATWADPNSTTEEAATQKEKWFENEWQGKNPPTPSEDSHVT
EGTT 265
3DWL_A : 183 VVDSGDGVTHIIPVAEGYVIGSSIKT-
MPLAGRDVTYFVQSLLR########LKTAERIKECCYVCPDIVKEFSRFDR#####LKYA 269
              * **       *  ***       *  *   **   *  **
*   **         *
similarity= 18/85 = 21.18%

CD44v6 : 181 DVSSGSTIEKSTPEGYILHTDLPTSQPTGD-----
RDDAFFIGSTLATWADPNSTTEEAATQKEKWFENEWQGKNPPTPSEDSHVTEGTT 265
3LQ1_A : 276
FLKEAEIIDKLTPEVVIRFGSMPVSKPLKNWLEQLSDIRFYVVDPGAAWKDPIKAVTDMIHCDERFLL
DIMQ-QNMPDDAKDAAWLNGWT 364
                    * * ***  *     * * *         *  *      * * **
*       *  * *    *     * *

similarity= 22/85 =25.88%
```

**Figure S1** Representative sequence comparison results obtained from BLAST server. An * (asterisk) indicates positions which have a single, fully conserved residue.

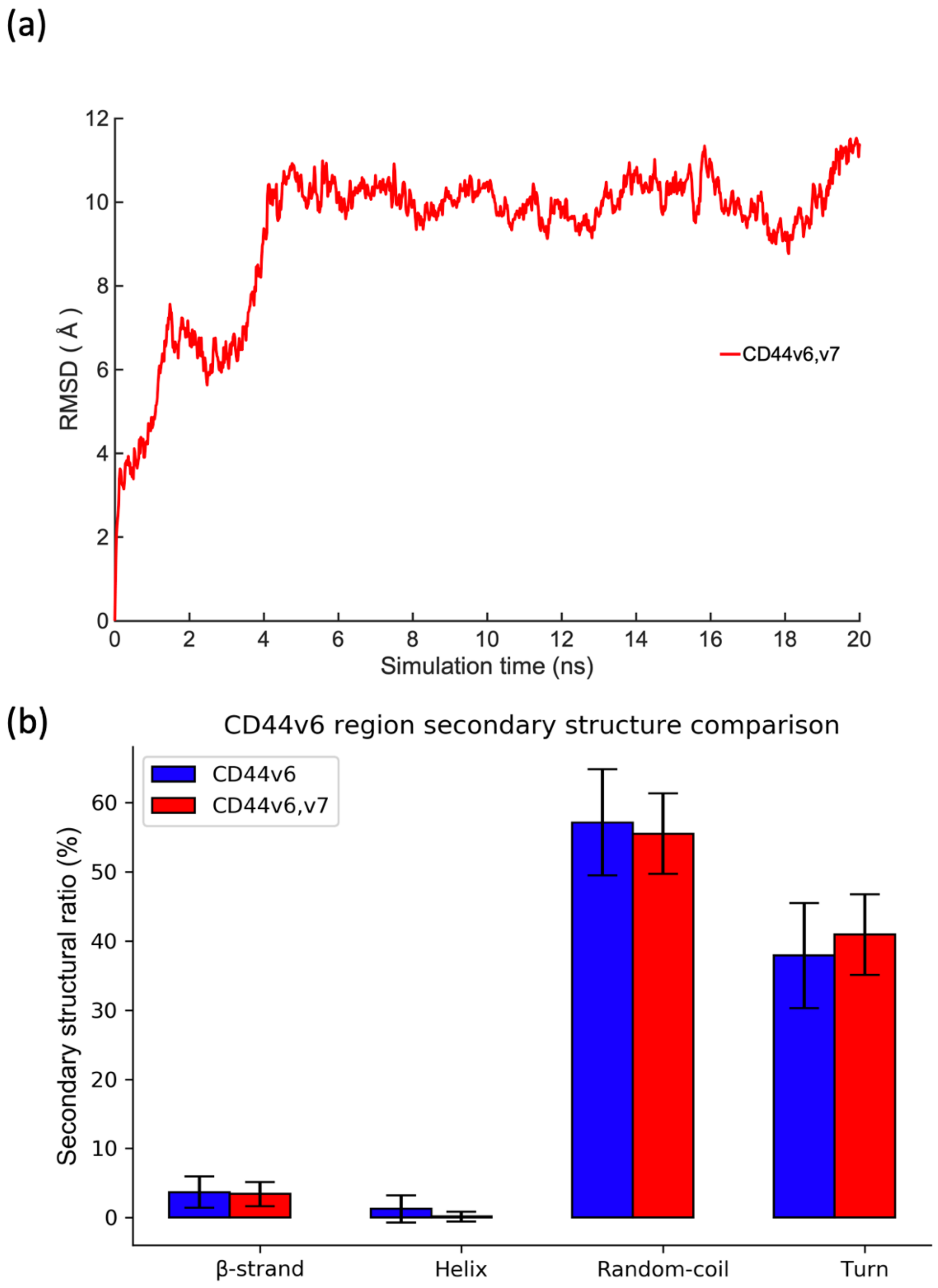


**Figure S2** (a) The RMSD of CD44v6+v7 and (b) secondary structure comparison between CD44v6 and CD44v6+v7.

**Table S1.** Hydrogen bond occupancies for CD44s cluster 1. The hydrogen bonds involved in β-sheets were underlined. (Only occupancies above 35% are shown.)

| Donor | Acceptor | Occupancy (%) |
|---|---|---|
| HIS199-Main-N | **PHE215-Main-O** | 88.60% |
| ILE217-Main-N | ILE197-Main-O | 81.58% |
| GLY195-Main-N | THR192-Main-O | 64.04% |
| THR192-Main-N | TYR196-Main-O | 50.88% |
| TYR196-Main-N | THR192-Side-O | 50.00% |
| THR187-Main-N | THR200-Main-O | 43.86% |
| PHE215-Main-N | HIS199-Main-O | 42.98% |
| THR200-Main-N | THR187-Main-O | 40.35% |
| LEU202-Main-N | GLY185-Main-O | 39.47% |
| GLU194-Main-N | THR192-Side-OG1 | 35.96% |
| GLU189-Main-N | LEU198-Main-O | 35.09% |

**Table S2.** Hydrogen bond occupancies for CD44s cluster 2 The hydrogen bonds involved in β-sheets were underlined. (Only occupancies above 35% are shown.)

| Donor | Acceptor | Occupancy (%) |
|---|---|---|
| ILE217-Main-N | SER183-Main-O | 66.67% |
| SER183-Main-N | **PHE215-Main-O** | 65.71% |
| LEU202-Main-N | THR200-Side-O | 51.43% |

**Table S3.** Hydrogen bond occupancies for CD44v6 cluster 1. The hydrogen bonds involved in β-sheets were underlined. (Only occupancies above 35% are shown.)

| | Donor | Acceptor | Occupancy (%) |
|---|---|---|---|
| Within standard stem | GLU194-Main-N | SER191-Main-O | 58.73% |
| | GLN206-Main-N | THR204-Side-O | 45.63% |
| | GLY209-Main-N | GLN206-Main-O | 42.46% |
| | LEU221-Main-N | GLY218-Main-O | 40.08% |
| | GLY195-Main-N | SER191-Side-O | 38.89% |
| | ALA214-Main-N | ARG211-Main-O | 38.89% |
| Between standard stem and v6 | TRP224-Main-N | **PHE215-Main-O** | 75.40% |
| | **PHE215-Main-N** | TRP224-Main-O | 63.10% |
| | TYR196-Main-N | ASP226-Main-O | 55.16% |
| Within variant 6 | GLU245-Main-N | PHE242-Main-O | 38.89% |
| | LYS238-Main-N | THR236-Side-O | 35.71% |

**Table S4.** Occupancies of hydrophobic and aromatic contacts with Phe215 (Only occupancies above 35% are shown.)

| | Hydrophobic/aromatic residue | Occupancy (%) |
|---|---|---|
| CD44s, cluster 1 | HIS199 | 63.2% |
| | ILE217 | 45.6% |
| CD44s, cluster 2 | VAL182 | 70.5% |
| | ILE217 | 60.0% |
| | PRO203 | 37.1% |
| CD44v6, cluster 1 | TYR196 | 68.7% |
| | TRP224 | 61.1% |
| | ILE197 | 40.1% |
| | ILE217 | 40.1% |

**Table S5** Hydrogen bond occupancies for CD44v6+v7 between standard stem and v6

| | Donor | Acceptor | Occurancy (%) |
|---|---|---|---|
| Between standard stem and v6 | TRP224-Main-N | PHE215-Main-O | 96.71 |
| | PHE215-Main-N | TRP224-Main-O | 82.73 |
| | TYR196-Main-N | ASP226-Main-O | 98.3 |
